# Identification of novel candidate target genes, including *EPHB3, MASP1 *and *SST *at 3q26.2–q29 in squamous cell carcinoma of the lung

**DOI:** 10.1186/1471-2407-9-237

**Published:** 2009-07-16

**Authors:** Ji Un Kang, Sun Hoe Koo, Kye Chul Kwon, Jong Woo Park, Jin Man Kim

**Affiliations:** 1 Department of Pathology, Columbia University Medical Center, New York, NY, USA; 2 Department of Laboratory Medicine, Chungnam National University College of Medicine, Taejeon, Republic of Korea; 3 Department of Pathology, Chungnam National University College of Medicine, Taejeon, Republic of Korea

## Abstract

**Background:**

The underlying genetic alterations for squamous cell carcinoma (SCC) and adenocarcinoma (AC) carcinogenesis are largely unknown.

**Methods:**

High-resolution array- CGH was performed to identify the differences in the patterns of genomic imbalances between SCC and AC of non-small cell lung cancer (NSCLC).

**Results:**

On a genome-wide profile, SCCs showed higher frequency of gains than ACs (*p *= 0.067). More specifically, statistically significant differences were observed across the histologic subtypes for gains at 2q14.2, 3q26.2–q29, 12p13.2–p13.33, and 19p13.3, as well as losses at 3p26.2–p26.3, 16p13.11, and 17p11.2 in SCC, and gains at 7q22.1 and losses at 15q22.2–q25.2 occurred in AC (*P *< 0.05). The most striking difference between SCC and AC was gains at the 3q26.2–q29, occurring in 86% (19/22) of SCCs, but in only 21% (3/14) of ACs. Many significant genes at the 3q26.2–q29 regions previously linked to a specific histology, such as EVI1,*MDS1, PIK3CA *and *TP73L*, were observed in SCC (*P *< 0.05). In addition, we identified the following possible target genes (> 30% of patients) at 3q26.2–q29: *LOC389174 *(3q26.2),*KCNMB3 *(3q26.32),*EPHB3 *(3q27.1), *MASP1 *and *SST *(3q27.3), *LPP *and *FGF12 *(3q28), and *OPA1*,*KIAA022*,*LOC220729*, *LOC440996*,*LOC440997*, and *LOC440998 *(3q29), all of which were significantly targeted in SCC (*P *< 0.05). Among these same genes, high-level amplifications were detected for the gene, *EPHB3*, at 3q27.1, and *MASP1 *and *SST*, at 3q27.3 (18, 18, and 14%, respectively). Quantitative real time PCR demonstrated array CGH detected potential candidate genes that were over expressed in SCCs.

**Conclusion:**

Using whole-genome array CGH, we have successfully identified significant differences and unique information of chromosomal signatures prevalent between the SCC and AC subtypes of NSCLC. The newly identified candidate target genes may prove to be highly attractive candidate molecular markers for the classification of NSCLC histologic subtypes, and could potentially contribute to the pathogenesis of the squamous cell carcinoma of the lung.

## Background

Lung cancer is responsible for the highest cancer-related morbidity and mortality worldwide [1]. Non-small cell lung cancer (NSCLC) comprises approximately 80% of all lung cancers; squamous cell carcinoma (SCC) and adenocarcinoma (AC) are the two most common subtypes of NSCLC [2]. Cumulative information suggests that the SCC and AC subtypes' progress through different carcinogenic pathways [2-4], but the genetic aberrations promoting such differences, especially for the molecular difference between two subtypes, remain unclear.

The most prevalent known chromosomal changes in NSCLC include gains/amplifications at 3q, 5p, 7p, and 8q, and losses at 3p, 8p, 9p, 13q, and 17p [5-7]. Many significant genes that map to these regions had previously been associated with specific histologies [2-5]. Gains of 3q, 7p, 12p, and 20q, as well as losses of 2q, 3p, 16p, and 17p, are more frequently detected in SCC, whereas gains of 1q and 6p as well as losses of 9q and 10p are more prevalent in AC [7-10]. One of the most prevalent and significant differences between SCC and AC, a gain at the chromosome 3q location, has been recognized in several molecular cytogenetic studies [3-5]. Emerging data suggests that regions of amplification of 3q have a profound effect on tumor development and house candidate biomarkers of disease progression, response to therapy, and prognosis of SCC [11]. These findings suggest that genes located at these chromosomal regions progress through differing pathogenic pathways, but the genetic aberrations promoting such differences are largely unknown.

Array CGH has been recognized as a successful and valuable tool for evaluation of the whole genome, as well as significant genetic information at the single gene level, and has enabled us to classify different neoplasm's based on characteristic genetic patterns [12]. It has been used extensively to study various human solid tumors including NSCLC [13-15]. Although, recurrent genetic alterations in NSCLC have been studied extensively, to our knowledge, only a few studies have been performed to date to correlate the molecular difference between histologic subtypes of NSCLC using high-resolution microarray CGH. Therefore, further investigations are needed to gain additional insight into the clinical significance of recurrent chromosomal alterations between the two subtypes of NSCLC.

In this study, therefore, we performed high-resolution array-CGH to compare the different patterns of genetics alterations, and to identify potential candidate genes that may be associated with phenotypic properties that differentiate early stage SCC from AC.

## Methods

### Tumor Samples and DNA Extraction

Twenty two SCCs and 14 ACs of the lung patients undergoing surgery as a primary treatment, without previous radiation or chemotherapy, were analyzed. This study has been reviewed and approved by the Institutional Review Board of the Chungnam National University Hospital. All cases were reviewed by pathologists to verify the original histopathological diagnosis, depth of tumor, invasion, tumor differentiation and lymph node metastasis. The written informed consent was obtained from each patient according to institutional regulations. The demographic and pathological data, including age, gender and the tumor stage were obtained by a review of the medical records. All of the patients were classified according to the WHO histologic typing of lung carcinomas and the UICC TNM (tumor-node-metastasis) staging system. Some of these samples were previously profiled for copy number variations [15].

Tumor preparations were performed as described previously [7]. DNA isolation was performed following the manufacturer's instructions (Promega, Madison, WI, USA), with some modifications as described before [15,16]: commercial genomic DNA was used as a reference (Human Genomic DNA: Female; Promega Corporation, Madison, WI; Cat. No. G1521).

### Contraction of BAC clones mediated array CGH microarray

The characteristics of the MacArray™ Karyo4000 chips (Macrogen, Inc., Seoul) [17-20] were used in this study consist of 4,046 human BACs, which were applied in duplicate and a resolution of 1 Mbp http://www.macrogen.co.kr. BAC clones were selected from the proprietary BAC library of Macrogen, Inc. All clones were two-end sequenced using an ABI PRISM 3700 DNA Analyzer (Applied Biosystems, Foster City, CA), and their sequences were blasted (using BLAST; http://blast.ncbi.nlm.nih.gov/Blast.cgi)) and mapped according to their positions, as described in the University of California, Santa Cruz (UCSC) human genome database http://www.genome.ucsc.edu (Build 36, Version Mar. 2006 (hg18)). Locus specificities of chosen clones were confirmed by removing multiple loci-binding clones individually under standard fluorescence *in situ *hybridization (FISH) [21]. These clones were prepared using the conventional alkaline lysis method to obtain BAC DNA. The arrays were manufactured using an OmniGrid arrayer (GeneMachine, San Carlos, CA) using a 24-pin format. Each BAC clone was represented on an array as triplicate spots and each array was pre-scanned using a GenePix 4200A scanner (Axon Instruments, Foster City, CA) for proper spot morphology.

### Array CGH experiment

Array CGH was performed as described previously [15]. Briefly, arrays were pre-hybridized with salmon sperm DNA to block repetitive sequences in the BACs. 500 ng of normal male DNA (reference) and digested tumor DNA (test) were labeled with Cy5-dCTP and Cy3-dCTP, respectively, by randomly primed labeling (Array CGH Genomic Labeling System; Invitrogen, CA, USA). The labeled probe and human Cot-I DNA were mixed and dissolved in hybridization solution. Hybridizations were performed in a sealed chamber for 48 h at 37°C. After hybridization, array slides were scanned on a GenePix 4200A two-color fluorescent scanner (Axon Instruments, Union City, CA, USA); quantification was performed using GenePix software (Axon Instruments).

### Analyzed BAC clones

After scanning, the fluorescent intensities of the red and green channels were saved as two TIFF image files and the background was subtracted from these. Log_2_-transformed fluorescence ratios were calculated from background-subtracted median intensity values, and these ratios were used for normalization using intensity normalization methods. To adjust for effects due to variation between the red and green dyes, LOWESS normalization was applied. Then, the ratio of the red/green channel of each clone was calculated and log2 transformed. Spot quality criteria were set as foreground to background greater than 3.0 and standard deviation of triplicates less than 0.2. The breakpoint detection and status assignment of genomic regions is performed by the GLAD software was used [22]. The total number of 3,776 BAC clones was analyzed excluding the 31 missing values and sex chromosomes (238) since female tumor DNA was hybridized with male control DNA to serve as an internal control. A low-level copy number gain was defined as a log 2 ratio > 0.25 and a copy number loss was defined as a log 2 ratio <-0.25. High-level amplification of clones was defined when their intensity ratios were higher than 0.8 in log_2 _scale and vice versa for homozygous deletion [23-25]. This threshold value was defined empirically as a value 3-fold that of the standard deviation calculated from 30 normal male to normal females in hybridization experiments. Macrogen's MAC viewer v1.6.6, CGH- explorer 2.55, and avadis 3.3 prophetic were used for graphical illustration and image analysis of array CGH data.

### Statistical analysis for array CGH

For group comparison, the differences in log2 ratios, as well as the Fisher exact test were used to determine whether there was any significant gain or loss of genomic content within particular cytobands with cancer type. The Fisher exact test utilized two categories normal and abnormal (loss and gain), with the null hypothesis that the relative proportions of each of the two imbalance categories would be expected to be the same in the groups. The counts of abnormal versus normal were summarized by subtype of NSCLC (SCC and AC)for each BAC, providing 2 × 2 tables for analysis. A multiple testing correction (Benjamini-Hochberg false discovery rate (FDR)) applied to correct for the high number of false positive calls. The R 2.2.1 package of the Bioconductor Project http://www.bioconductor.org was used for detection of the frequency of gain or loss and statistical analysis. Macrogen's MAC viewer v1.6.6, CGH- explorer 2.55, and avadis 3.3 prophetic were used for graphical illustration and image analysis of array CGH data.

### Quantitative Real-time PCR analysis

Real-time quantitative PCR analysis was performed using the ABI PRISM 7900HT Sequence Detection system and TaqMan Gene Expression assays according to the manufacturer's instructions (Applied Biosystems, Foster city, CA). In brief, samples (2.5% of the reverse transcription reaction) were amplified using the Universal Master Mix (Applied Biosystems) and cycling conditions of 15 s of denaturating time (95°C) and 1 min of annealing/amplification time (60°C) for 40 cycles after an initial activation step of 10 min at 95°C. All samples were assayed in triplicates. To enable detection of possible contaminating genomic DNA, we analyzed non-reversed transcribed total RNA from all tumors in parallel with the cDNAs. Normalized normal human pooled genomic DNAs (Promega, Madison, WI, USA) were used as reference DNAs. All data analysis was used ArrayAssist^® ^(Stratagene, La Jolla, USA) and R (Ver.2.7.2). Correlation between BAC chip and Q RT-PCR data was performed by Pearson correlation analysis (*P *< 0.05).

## Results

### Array CGH analysis in SCCs and ACs of the lung

**Figure 1:**
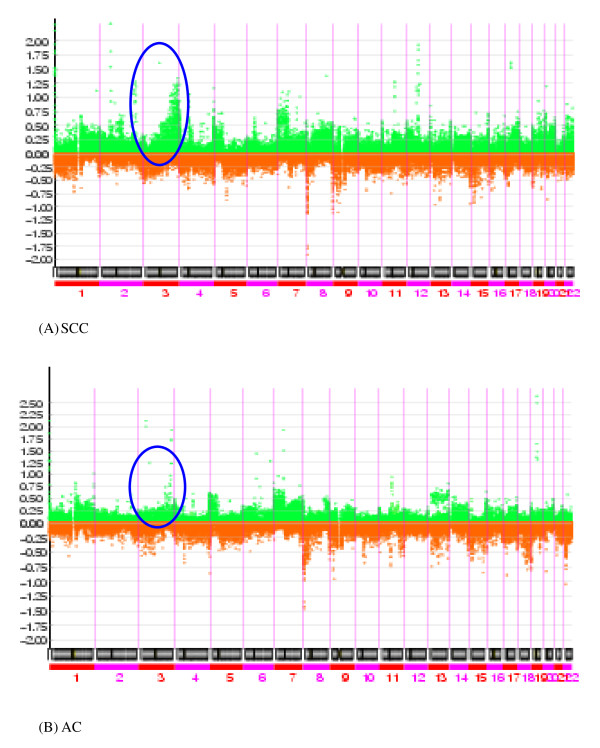
**The X-axis represents chromosome number (1 – 22) and the Y-axis represents the genome-wide frequencies of gains (> 0.25 of intensity ratio) and losses (< 0.25 of intensity ratio) for each clone of NSCLC are shown**. Vertical lines indicate chromosomal boundaries. (A) Frequencies of genes increased in copy number or decreased in copy number in SCC of the lung. (B) Frequency of gene copy number abnormalities in the AC. Gains and losses are shown as green and red color bars, respectively, which indicate the overall chromosomal copy number aberrations detected.

**Figure 2:**
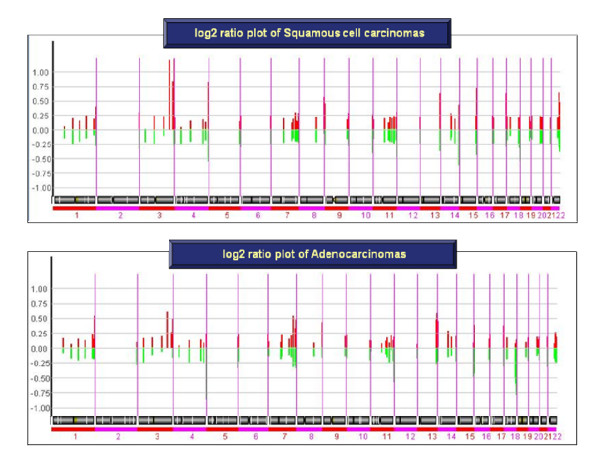
**Signal intensity ratios for each spotted BAC clones of SCCs and ACs displayed as log2 plots**. A total of 4,046 BAC clones were ordered (x-axis) according to the map positions and the chromosomal order from 1pter to 22qter.

One-megabase through put whole genome array-CGH was performed to establish distinct differences in chromosomal copy number changes between the SCC and AC histologic subtypes of NSCLC. Clinicopathological data for the 22 SCCs and 14 ACs patients are summarized in Table 1. All of the NSCLC patients (100%) had copy number aberrations and each patient evidenced numerous copy number changes. On average, 173 clones were gained (range, 14–579), and 136 clones were lost (range, 5–537) per patient. Although the difference in the copy number gain, between the two histologic subtypes was not statistically significant, we found higher frequency of gains in SCC compared to AC (203 vs. 125, respectively, *P *= 0.067). To visualize both common and specific altered chromosomal regions in each subtype of NSCLC, signal intensity ratios for each spotted BAC clones were calculated and displayed as log2 plots (Figures 1 and 2). Most of the chromosomes in this profile showed multiple segmental alterations, including single copy as well as high level gains and losses.

**Table 1:** Summary of clinico pathological data of the samples

				TNM classification	
				
No	Gender	Age	Histology	Lymph node status	Tumor stage
1	M	61	AC	Absent	IB
2	F	50	AC	Present	IIIA
3	F	47	AC	Absent	IB
4	M	66	AC	Absent	IA
5	M	65	AC	Present	IIIA
6	F	61	AC	Absent	IA
7	F	56	AC	Present	IIB
8	M	72	AC	Absent	IA
9	M	61	AC	Present	IIB
10	F	70	AC	Present	IIIA
11	M	60	AC	Present	IIIA
12	M	70	AC	Present	IIB
13	M	75	AC	Present	IIIA
14	F	69	AC	Absent	IA
15	M	67	SCC	Present	IIA
16	M	51	SCC	Present	IIIA
17	M	59	SCC	Absent	IB
18	M	69	SCC	Absent	IB
19	M	62	SCC	Absent	IA
20	F	72	SCC	Absent	IB
21	M	60	SCC	Present	IIIA
22	M	64	SCC	Absent	IB
23	M	65	SCC	Absent	IB
24	M	65	SCC	Present	IIB
25	M	64	SCC	Present	IIB
26	M	65	SCC	Present	IIB
27	M	64	SCC	Absent	IB
28	M	64	SCC	Absent	IB
29	M	66	SCC	Present	IIIA
30	M	74	SCC	Absent	IB
31	F	72	SCC	Absent	IA
32	M	74	SCC	Absent	IB
33	M	69	SCC	Absent	IB
34	M	55	SCC	Present	IIIA
35	M	90	SCC	Present	IIB
36	F	69	SCC	Absent	IA

### Distinct genomic signatures between SCC and AC histologic subtypes

**Figure 3:**
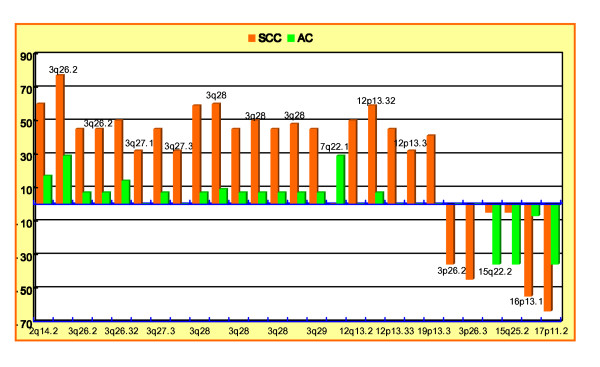
**The statistically significant genomic regions preferentially gained and lost between SCC and AC of NSCLC patients**. Significant genomic regions between SCC and AC of NSCLC represent in the x-axis and the percentage of gains (upper panel) and losses (lower panel) expressed regions in each chromosomal regions is illustrated to the y-axis (red bars represent the SCC and green bars represent ACs).

Across the whole genome, we identified specific genomic alterations between the two subtypes. Gains at 2q, 3q, 12p, and 19p, as well as losses at 3p, 4p, 16p, and 17p were found specific to SCC, whereas gains at 6p and 7q and losses at 4q and 15q were more prevalent in AC. A summary of preferentially gained and lost genomic changes for patients with SCC and AC are listed in Table 2 (> 30% of patients). More specifically, statistically significant differences were observed across the histologic subtypes. We identified significant chromosomal regions between the two subtypes for gains of 2q14.2, 3q26.2–q29, 12p13.2–p13.33, and 19p13.3, as well as losses of 3p26.2–p26.3, 16p13.11, and 17p11 in SCC (P < 0.05), and a gain of 7q22.1 and losses of 15q22.2–q25.2 in AC (P < 0.05). The statistically significant genomic regions preferentially gained and lost by histologic subtypes and the potential target genes are summarized in Table 3 and Figure 3 (see Additional file 1).

**Table 2:** Summary of histology-enriched chromosomal alterations by array CGH analysis

Chromosomal alterations	Histology	
	
	Squamous cell carcinomas	Adenocarcinomas
Gained regions	1p36.33-1q44	1p36.33-1q44
	2q14.2	5p13.2–5p15.33
	3q13.12–3q29	6p25.3
	5p12–5p15.33	7q22.1–7q36.3
	7p12.2–p22.3	16p13.3
	9q34.3	17q21.2–17q25.3
	10q26.3	20q13.33
	11p13.3-11q15.5	
	12p11.1-12q24.33	
	13q21.1–13q34	
	15q22.2–q26.3	
	16p13.3-16q24.3	
	17q23.2–17q25.3	
	19p13.3–19q12	
	20q13.33	

Lost regions	1p21.1–1p36.33	1q21.1
	2q37.1	4q13.3
	3p12.2–3p26.3	5q13.2
	4p12–p16.1	8p12–8p23.3
	5q11.2–5q35.1	13q12.11–13q21.1
	8p21.2–8p23.3	14q32.33
	9p12–p13.1	15q11.2–15q25.3
	10q11.22–10q26.3	18q21.2
	13q12.11–13q14.3	19q13.2–19q13.31
	14q32.33	20p11.23
	16p12.3–16p13.11	21q11.2
	17p11.2–17p13.3	22q11.1
	18q12.3–18q23	
	20p11.23	
	21q11.2	
	22q11.1	

**Table 3:** BAC clones with significant frequency differences between SCC and AC subtypes of NSCLC

BAC clone	Regions	Gene contained in clones	% of gain in SCCs^‡^	% of gain in AC^†^	p-value*
**Gains^¶^**					
BAC13_I04	2q14.2		**55**	7	0.046
BAC215_M07	3q26.2	*EVI1*	**77**	29	0.007
BAC28_H10	3q26.2	*MDS1*	**45**	7	0.034
BAC127_C12	3q26.32	*PIK3CA, KCNMB3*	**50**	14	0.050
BAC78_P09	3q26.2	*LOC389174*	**45**	7	0.034
BAC97_L20	3q27.1	*EPHB3*	**32**	0	0.039
BAC187_G13	3q27.3	*MASP1*	**45**	7	0.034
BAC195_F06	3q27.3	*SST*	**32**	0	0.039
BAC62_G09	3q28		**64**	7	0.002
BAC29_O18	3q28	*TP73L*	**45**	7	0.034
BAC74_E06	3q28		**50**	7	0.015
BAC143_F11	3q28	*FGF12*	**45**	7	0.034
BAC144_N20	3q28	*LPP*	**48**	0	0.032
BAC95_M19	3q29	*OPA1*	**55**	7	0.046
BAC96_C05	3q29	*KIAA0226, LOC220729, LOC440996, LOC440997, LOC440998*	**45**	7	0.034
BAC42_D19	7q22.1	*TRRAP*	0	**29**	0.023
BAC141_P01	7q22.1	*ZKSCAN1, ZNF38, ZNF3*	0	**29**	0.023
BAC118_C23	12p13.2	*CDKN1B, DKFZP434F0318*	**50**	0	0.003
BAC26_P09	12p13.32	*TSPAN9, LOC399986, LOC441627*	**59**	7	0.001
BAC159_B18	12p13.33	*LOC440123, SLC6A12, SLC6A13*	**45**	0	0.002
BAC179_J19	12p13.33	*SLC6A12, SLC6A13*	**32**	0	0.003
BAC18_B14	19p13.3	*SCHC2, C19orf19, MADCAM1*, *C19orf20, CDC34, GZMM, BSG*	**41**	0	0.008
					
**Losses**^§^					
BAC83_J18	3p26.2	*LOC440943*	**36**	0	0.018
BAC229_D19	3p26.3	*CCR2, CCRL2, APOD*	**45**	0	0.005
BAC57_A15	15q22.2	*RORA*	5	**36**	0.033
BAC175_C05	15q25.2	*DNM1DN11-6, LOC388160, LOC440300, LOC388152, LOC161527, LOC440301, LOC159170*	5	**36**	0.033
BAC169_L10	16p13.11		**55**	7	0.046
BAC130_G23	17p11.2		**64**	36	0.039

Abbreviations: ^†^AC, Adenocarcinoma; ^‡^SCC, Squamous cell carcinoma; * *P *values from Fisher's exact test on categorized log2 ratio values. ^¶^Alterations were defined by log2 ratio thresholds of 0.25 for copy number gain. Using this threshold, we generated a frequency table. ^§^Alterations were defined by log2 ratio thresholds of – 0.25 for copy number loss. Using this threshold, we generated a frequency table.

Significant differences were not reached for gains at 3q29 (*LMLN*, *LOC348840*,*LOC442100*, and *BDH*; *P *= 0.085) or losses at 3p25.1 (*ZFYVE20 *and *LOC344875*; *P *= 0.084), 3p26.1 (*GRM7*; *P *= 0.084), 3p26.3 (*CHL1*; *P *= 0.084) and 4p16.1 (*WFS *and *PPP2R2C*; *P *= 0.084) in the SCC series, and gains at 6p25.3 (*LOC44118*; *P *= 0.065), 7q21.11 (*CD36*; *P *= 0.069), 7q32.1 (*SND1*; *P *= 0.069), 7q34 (*EPHB6, TRPV6, TRPV5, C7orf34 *and *KEL*; *P *= 0.069) or losses at 4q13.3 (*ADAMTS3*; *P *= 0.069) in the AC group; however, all had borderline significances (data not shown).

### Significant copy number differences of target genes at 3q26.2–q29 between SCC and AC

**Figure 4:**
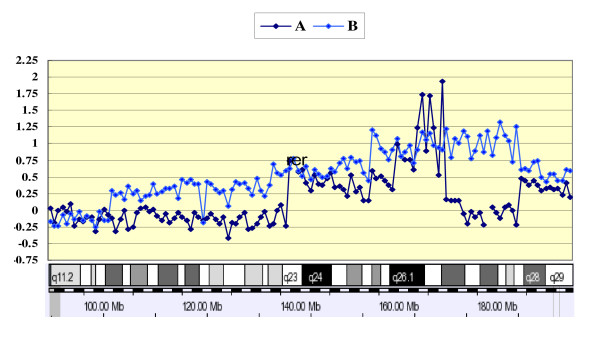
**Individual profiles of high copy number changes at 3q**. A. High-level amplifications on 3q27 for AC from patient 12, B. 3q26.2–q28 for SCC from patient 27. In the intensity ratio profiles, the x-axis represents the map position of corresponding clone and the intensity ratios were assigned to the y-axis. The schematic presentation of cytogenetic bands as well as a map position is shown below the plot.

In this array profile, the most striking difference between SCC and AC was gains at 3q26.2–q29, with 19 of 22 patients showing gains in at least part of these chromosomal regions (86%) in SCCs, but only 21% (3/14) were observed in ACs. Several putative cancer-related genes at the 3q26.2–q29 regions were previously linked to specific histologic subtypes; specifically, gains at EVI1 and *MDS1 (3q26.2*),*PIK3CA *(3q26.32), and *TP73L *(3q28) were significantly observed in SCCs (*P *< 0.05). Additionally, we identified possible candidate target genes in these chromosomal regions, that are not yet known for their involvement in the pathogenesis of SCC (> 30% of patients): namely, *LOC389174 *(3q26.2),*KCNMB3 *(3q26.32),*EPHB3 *(3q27.1), *MASP1 *and *SST *(3q27.3), *LPP *and *FGF12 *(3q28), and *OPA1, KIAA022, LOC220729, LOC440996, LOC440997 *and *LOC440998 *(3q29), all of which were significantly targeted in SCC (*P *< 0.05). A representative high level amplification observed recurrently at 3q27 (*MCF2L2 *and *B3GNT5*) in AC (A), and an example of an individual profile of high level amplifications around the 3q26.1–q28 regions in SCC (B) is presented in Figure 4.

Among these significantly associated genes at 3q26–q29 in SCC, high-level amplifications were detected for the genes, *EVI1 *and *MDSI *at 3q26.2 (23 and 5%, respectively), and *EPHB3*,*MASP1*, and *SST *at 3q27.1–q27.3 (18, 18, and 9%, respectively). For this analysis, we defined a high level amplification as log2 signal intensity ratio reaching +0.8.

**Figure 5:**
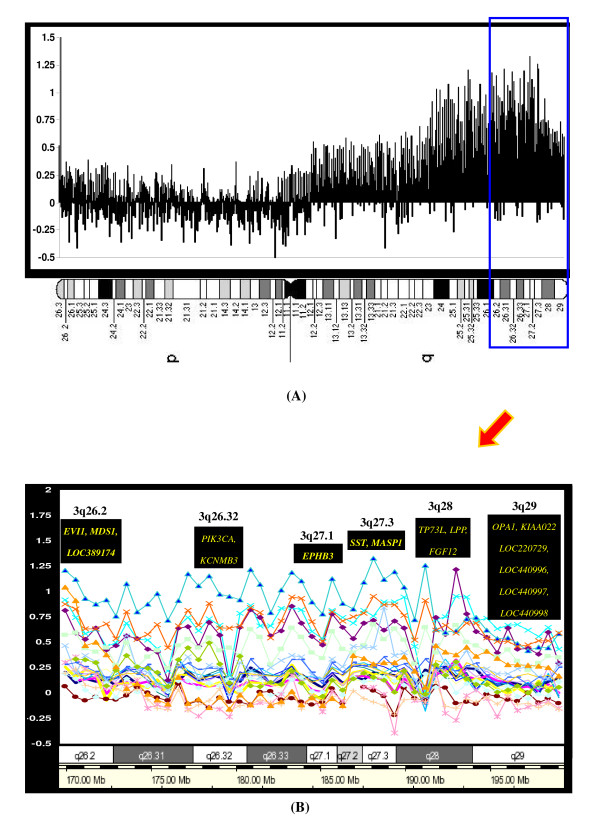
**(A) Weighted frequency (%) diagram for chromosome 3 with high-level amplifications**. In the intensity ratio profiles, the x-axis represents the map position of the corresponding clone and the intensity ratios were assigned to the y-axis. (B) More detailed profile on 3q26.2–q29 with the significantly associated genes in the SCC. The schematic presentation of cytogenetic bands as well as a map position is shown below the plot.

As a gain of *EVI1 *and *MDSI *at 3q26.2 has been described previously in SCC, we sought to determine whether there exist any correlations between *EVI1 *or *MDSI *and the newly amplified genes of *EPHB3, MASP1*, or *SST. Interestingly, c*o-amplifications were demonstrated for *EVI1 *and *EPHB3 *in 18% and for *EVI1 *and *SST *in 14%. All of the amplified genes, including significantly associated targets in SCC at the 3q26.2–q29 regions, are summarized in Table 4. Figure 5A shows a weighted frequency (%) diagram for chromosome 3 with high-level amplifications, and Figure 5B represents more detailed profiles at the 3q26.2–q29 regions with the significantly associated genes in SCC. The data discussed in this publication have been deposited in NCBIs Gene Expression Omnibus (GEO) and are accessible through GEO Series accession number GSE 16597 http://www.ncbi.nlm.nih.gov/geo/query/acc.cgi?acc=GSE16597.

**Table 4:** High-level amplifications at 3q26.2–q29 by array CGH analysis in NSCLC, together with candidate genes

BAC clone	Chromosome location	Gene contained in clones	% of case of amplifications
**SCC**^‡^			
BAC215_M07	3q26.2	*EVI1*	23 (5/22)
BAC28_H10	3q26.2	*MDSI*	5 (1/22)
BAC130_O18	3q26.31	*NAALADL2*,	9 (2/22)
BAC1_N14	3q26.32		9 (2/22)
BAC231_I11	3q26.33	*LOC401102*,	14 (5/22)
	3q27.1	*EPHB3*,	18 (4/22)
BAC187_G13	3q27.3	*MASP1*	18 (4/22)
BAC179_E01	3q27.3	*BCL6, LOC389185*	14 (3/22)
BAC195_F06	3q27.3	*SST*	14 (3/22)
BAC74_E06	3q28	*CLDN1*,	9 (2/22)
BAC144_N20	3q28	*LPP*	9 (2/22)
**AC**^†^			
BAC176_G15	3q27.1	*MCF2L2, B3GNT5*	7 (1/14)

Abbreviations: Abbreviations: ^‡^SCC, Squamous cell carcinoma; ^†^AC, Adenocarcinoma.

### Real Time quantitative PCR analysis

To validate the consequences of gene amplification by array CGH, we performed subsequently real-time quantitative PCR analyses for three potential oncogenes (*EPHB3, MASP1*, or *SST*) at 3q27.1–q27.3. Primers for the three genes (*EPHB3, MASP1*, or *SST) are presented in *Table 5.

**Table 5:** Primers used for Real-time quantitative PCR analysis

Gene name	Chromosome location	Primer forward	Primer reverse
EPHB3	3q27.1	GGGTCAGAACCTTCCCTAAAGTG	GTTAAAGATACCCCAAATAAGTACTGCCT
MASP1	3q27.3	CCTTGCAAGATGAGGGAGTTCAG	GCCCCTATACTCTTCCTTCCTATGT
SST	3q27.3	TGACCGACTGCGCTTATCATG	TCCCGAAGCTCTTGAGAAAACTATC

**Figure 6:**
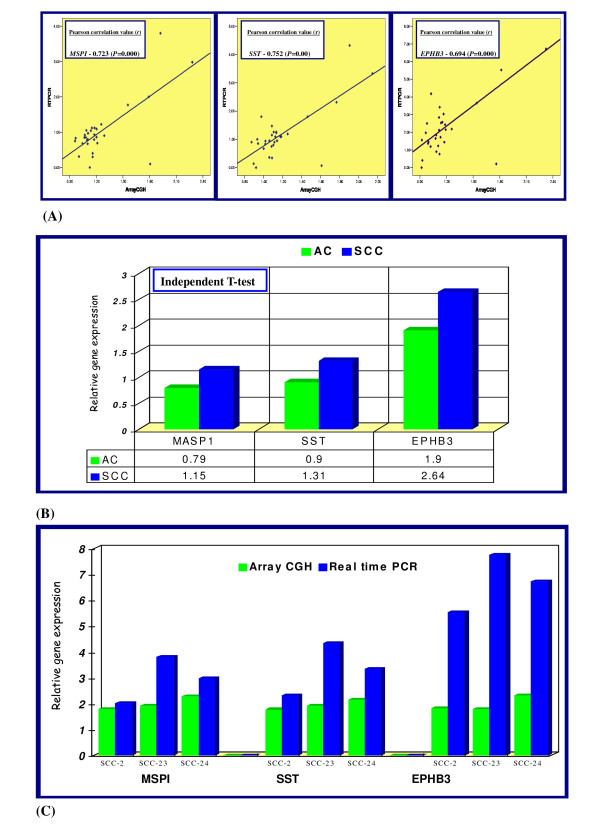
**(A) The scatter plot analysis of all data points of three genes (*EPHB3, MASP1 *and *SST*) by Array CGH (*X axis*) and Q-RT-PCR (*Y axis*) analysis in NSCLC patients**. Each dark square, the log2 ratio value of the clone containing one of these genes for one case. The correlation coefficients (**r**) and *P*-value are given in the upper left corner of the figure. (B) Comparison of real time PCR analysis of *EPHB3, MASP1*, or *SST *between SCCs and ACs. Note that the mean expression levels for selected genes were higher in SCCs compared to ACs. (C) Gene expression levels in Q RT PCR of three genes in three cases seen with amplification at 3q27.1–q27.3 by Array CGH. Each samples are depicted (*x*-axis) and the fold difference of array CGH was depicted by linear-ratios, and *N*-value was delineated in real time PCR (*y*-axis). The heights of the bars represent mean expression level of the indicated genes in each SCC cases.

Although the absolute values of selected genes were different between two analyses, significant correlations were observed between two data sets (*P *< 0.05). The value of array CGH was depicted by linear-ratios and *N*-value was delineated in real time PCR. Correlation coefficients between gene expression levels in real time PCR and array CGH analysis for three genes (*EPHB3, MASP1*, or *SST*) were 0.694, 0.723 and 0.752, respectively. Figure 6A shows a comparison of mean relative expression levels for *EPHB3, MASP1*, or *SST *genes between array CGH and real time PCR results in NSCLCs and three cases with amplification at 3q27.1–q31.3 are shown in Figure 6C. Moreover, real time PCR analysis demonstrated higher expression levels for *EPHB3, MASP1*, and *SST *genes in SCCs compare to ACs. This corresponds are shown in Figure 6B.

## Discussion

Non-small cell lung cancer is the most frequently occurring type of lung cancer, with SCC and AC being the two main subtypes [12]. Since the most common genetic aberrations in NSCLC had already been identified in our previous study [15], we paid attention to explore candidate genes that may be associated with phenotypic properties that differentiate early stages SCC from AC. A whole genomic strategy allowed us to define candidate regions that may contain specific cancer-related genes involved in early stages of SCC and AC.

Gains of 2q, 3q, 12p, and 19p, as well as losses of 3p, 4p, 16p, and 17p, were found to be specific to SCC, whereas gains of 6p and 7q and losses of 4q and 15q were more prevalent in AC. More specifically, statistically significant differences were observed between the two subtypes for gains at 2q14.2, 3q26.2–q29, 12p13.2–p13.33, and 19p13.3, as well as losses at 3p26.2–p26.3, 16p13.11 and 17p11.2 in SCCs, whereas gains at 7q22.1 and losses at 15q22.2–q25.2 were observed in ACs (*P *< 0.05). These findings support the notion that the two histologic subtypes progress through different pathogenic pathways and further indicate that SCC and AC can be readily distinguished from each other at the chromosomal level.

Although, these findings are similar to the findings reported in previous studies [26-29], four chromosomal regions for gains at 2q14.2 or 19p13.3 in SCC, and gains at 7q22.1 or losses at 15q22.2–q25.2 in AC, have not been described as focal regions of lung cancer.

We found 55% of copy number gains at the 2q14 region in SCCs, but only 7% of gains were detected in ACs (*P *= 0.046). Although, little is known about gains at chromosome 2q14, the genomic region containing this region may harbor potential oncogenes involved in the tumorigenesis of SCC. Future studies will be needed to verify the significances of this outcome.

A gain at 19p13.3 was observed in 41% of SCCs, whereas no gains in ACs (*P *= 0.008), which harbored the genes *SHC2*,*C19orf19*,*MADCAM1*,*C19orf20*,*CDC34*, *GZMM*, and *BSG*. Emerging data [30] have described that the *C19orf19 *gene product is *EGFR*-associated and phosphorylated at 5 tyrosines in response to *EGFR *activation and, therefore, represents a new component of the *EGFR *signaling network. The over expression of the EGFR gene in SCC has been well recognized in several molecular cytogenetic studies [8,31,32]. Although no significant correlation was found regarding the association between *EGFR *and *C19orf19 *in this study (data not shown), further investigations will enable us to determine the functional associations of these two genes, and whether these genes or additional genes at 19p13.3 contribute to the genome differentiating SCC from AC.

The present study revealed that gain of 7q22.1 is more specific for lung AC than for SCC (*P *= 0.023). This region detected as an 89-kbp gene-specific copy number gain, entered at *TRRAP*,*ZKSCAN1*,*ZNF38 *and *ZNF3*. Loukopoulos et al. [33] recently showed that the frequent amplification of the *TRRAP *gene in AC from the pancreas, and another study using array-CGH reported high level amplifications at 7q21–q22 in gastric ACs [34]. Taken together, these results and our own results suggest that this region might be affected in tumorigenesis of AC.

Chromosome 15q was detected in 36% of copy number losses with 15q22.2–q25.2 in the AC group, whereas only 5% of loss were detected in SCC (*P *= 0.033). These regions have not been described as a common change in AC of the lung thus far, but are commonly found in ACs from follicular and clear cell AC. Roque et al. [35] demonstrated that 15q loss was significantly associated with follicular adenocarcinomas, and Okada et al. [36] reported that the loss of heterozygosity (LOH) at 15q was detected in at least 50% of clear cell ACs, indicating that these candidate regions may contain specific cancer related genes involved in AC. Future work will validate of these findings.

In this array survey, the most salient discriminators between SCC and AC were gains at 3q26.2–q29, occurring in 86% of SCCs, whereas only 21% were observed in ACs. Furthermore, high-level amplifications in these regions were more prevalent in SCCs than ACs (6/22 = 27% vs. 1/14 = 7%, respectively). Our data also pointed out that 4 of 6 (67%) high level amplifications at 3q26.2–q29 regions were detected in stage (I+IIA) SCCs without lymph node metastatic lesions. It is very interesting to note that the high level amplifications at 3q26.2–q29 were more prevalent in stage (I+IIA) SCCs than more advanced stages. Heselmeyer *et al*. [37,38] reported that gain of chromosome 3q could be found in early dysplasic lesions as well as in invasive cervical cancer, but at a reduced frequency in advanced stages of disease. Furthermore, Yen *el al*. [39] described that the high-level amplifications on 3q25.3-qter were all found in stage IB tumors in esophageal squamous cell carcinoma. By combining results of this study with other reports, it is very likely that amplification of genes located on 3q may occur in early stages in the cancer. One possible explanation is that the alterations of genes are no longer necessary for maintenance of cancer cells survival [40]. Further studies are needed to confirm this hypothesis.

Previous analyses of the NSCLC genome with low-resolution chromosomal or BAC array-CGH have consistently demonstrated genome differentiation between SCC and AC in the telomeric subregion, 3q26-qter [40-43]. Several interesting cancer-related genes located in these genomic alteration regions have previously been identified with specific histologies, such as EVI1 (ecotropic viral integration site 1) and *MDS1 (*myelodysplastic syndrome 1) at 3q26.2,*PIK3CA *(phosphoinositide-3-kinase, catalytic, alpha polypeptide) at 3q26.32, and *TP73L *(tumor protein p73-like) at 3q28 region [40,41]. In this array survey, we sought to determine whether there exist additional candidate genes at 3q26-qter regions that drive genome differentiation of SCC from AC subtypes of NSCLC, and we were capable of detecting the following possible target genes previously not assumed to play a pathogenic role in SCC; *LOC389174 *(3q26.2),*KCNMB3 *(3q26.32),*EPHB3 *(3q27.1), *MASP1 *and *SST *(3q27.3), *LPP *and *FGF12 *(3q28), and *OPA1*,*KIAA022*, *LOC220729*, *LOC440996*,*LOC440997*, and *LOC440998 *(3q29), all of which were significantly targeted in SCC (*P *< 0.05). These genes have not been described in squamous cell carcinoma of the lung thus far, but are commonly found in other cancers or cancer cell lines [40-44]. Lukashova-v Zangen I *el al*. [43] described that the overexpression of *EPHB3 *gene in ependymomas with high proliferation indices was associated with a poor outcome, and Kuraya *el al*. [45] demonstrated the high expression of *MASP1 *gene in glioma cell line. More strikingly, among these possible target genes within 3q26–q29 regions, *EPHB3 *(3q27.1), and *MASP1 *and *SST *(3q27.3) showed high-level amplifications, in more than three patients each in SCC, implicating that these genes may be major potential targets for characterization of NSCLC histologic subtypes. Real time PCR analysis demonstrated over expression level for the three genes (*EPHB3*, *MASP1 *and *SST*) in SCCs compare to ACs. These results were in agreement with array-CGH results.

## Conclusion

The high-resolution analysis allowed us to propose novel candidate target genes that may be associated with phenotypic properties that differentiate early stages of SCC from AC. The newly identified candidate genes could be useful biomarkers for the early detection and characterization of NSCLC histological subtypes as well as novel targets for therapeutic interventions of early stages of squamous cell carcinoma of the lung.

## Competing interests

The authors declare that they have no competing interests.

## Authors' contributions

JUK performed the experiments, data analysis, and wrote the manuscript. SHK contributed to the design of the study and critically reviewed the manuscript. KCK was involved in reviewing the manuscript and supervision of the study. JWP participated in data analysis and its interpretation. JMK provided the material obtained from Korea and participated in case selection and pathologic review. All authors have read and approved the manuscript.

## Pre-publication history

The pre-publication history for this paper can be accessed here:

http://www.biomedcentral.com/1471-2407/9/237/prepub

## Supplementary Material

Additional file 1 **The physical position of each BAC**. The data provided represent the physical position of each BAC start and stop points. Click here for file

## References

[B1] Parkin DM Bray FI Devesa SS Cancer burden in the year 2000. The global picture Eur J Cancer 2001 37 S4 66 10.1016/S0959-8049(01)00267-2 11602373

[B2] Sy SM Wong N Lee TW Tse G Mok TS Fan B Pang E Johnson PJ Yim A Distinct patterns of genetic alterations in adenocarcinoma and squamous cell carcinoma of the lung Eur J Cancer 2004 40 1082 94 10.1016/j.ejca.2004.01.012 15093586

[B3] Yakut T Schulten HJ Demir A Frank D Danner B Egeli U Gebitekin C Kahler E Gunawan B Urer N Oztürk H Füzesi L Assessment of molecular events in squamous and non-squamous cell lung carcinoma Lung Cancer 2006 54 293 301 10.1016/j.lungcan.2006.08.011 17011066

[B4] Jiang F Yin Z Caraway NP Li R Katz RL Genomic profiles in stage I primary non small cell lung cancer using comparative genomic hybridization analysis of cDNA microarrays Neoplasia 2004 6 623 35 15548372 10.1593/neo.04142 PMC1531667

[B5] Yokoi S Yasui K Iizasa T Imoto I Fujisawa T Inazawa J TERC identified as a probable target within the 3q26 amplicon that is detected frequently in non-small cell lung cancers Clin Cancer Res 2003 15 4705 13 14581340

[B6] Ubagai T Matsuura S Tauchi H Itou K Komatsu K Comparative genomic hybridization analysis suggests a gain of chromosome 7p associated with lymph node metastasis in non-small cell lung cancer Oncol Rep 2001 8 83 8 11115574 10.3892/or.8.1.83

[B7] Kang JU Koo SH Kwon KC Park JW Shin SY Kim JM Jung SS High frequency of genetic alterations in non-small cell lung cancer detected by multi-target fluorescence in situ hybridization J Korean Med Sci 2007 22 47 51 10.3346/jkms.2007.22.S.S47 PMC2694384 17923754

[B8] Chujo M Noguchi T Miura T Arinaga M Uchida Y Tagawa Y Patterns of chromosomal imbalances in Comparative genomic hybridization analysis detected frequent overrepresentation of chromosome 3q in squamous cell carcinoma of the lung Lung Cancer 2002 38 23 9 10.1016/S0169-5002(02)00151-4 12367789

[B9] Kang JU Koo SH Kwon KC Park JW Jung SS Gain of the EGFR gene located on 7p12 is a frequent and early event in squamous cell carcinoma of the lung Cancer Genet Cytogene 2008 184 31 7 10.1016/j.cancergencyto.2008.03.002 18558286

[B10] Massion PP Kuo WL Stokoe D Olshen AB Treseler PA Chin K Chen C Polikoff D Jain AN Pinkel D Albertson DG Jablons DM Gray JW Genomic copy number analysis of non-small cell lung cancer using array comparative genomic hybridization: implications of the phosphatidylinositol 3-kinase pathway Cancer Res 2002 1 3636 40 12097266

[B11] Qian J Massion PP Role of chromosome 3q amplification in lung cancer. Role of chromosome 3q amplification in lung cancer J Thorac Oncol 2008 3 212 5 10.1097/JTO.0b013e3181663544 18317062

[B12] Lee GY Yang WI Jeung HC Kim SC Seo MY Park CH Chung HC Rha SY Genome-wide genetic aberrations of thymoma using cDNA microarray based comparative genomic hybridization BMC Genomics 2007 3 8 305 10.1186/1471-2164-8-305 PMC2082448 17764580

[B13] Kang JU Kang JJ Kwon KC Park JW Jeong TE Noh SM Koo SH Genetic alterations in primary gastric carcinomas correlated with clinicopathological variables by array comparative genomic hybridization J Korean Med Sci 2006 21 656 65 10.3346/jkms.2006.21.4.656 16891809 PMC2729887

[B14] Cathie Garnis1 William W Lockwood1 Emily Vucic1 Yong Ge1 Luc Lam WL High resolution analysis of non-small cell lung cancer cell lines by whole genome tiling path array CGH Int J Cancer 2006 118 1556 64 10.1002/ijc.21491 16187286

[B15] Kang JU Koo SH Kwon KC Park JW Kim JM Gain at chromosomal region 5p15.33, containing TERT, is the most frequent genetic event in early stages of non-small cell lung cancer Cancer Genet Cytogenet 2008 1 182 1 11 10.1016/j.cancergencyto.2007.12.004 18328944

[B16] Varella-Garcia M Gemmill RM Rabenhorst SH Lotto A Drabkin HA Archer PA Franklin WA Chromosomal duplication accompanies allelic loss in non-small cell lung carcinoma Cancer Res 1998 58 4701 7 9788625

[B17] Kim JI Lee JH Seo JS Hwang KT Han W Cho J Lee JW Ko E Kim EK Jung SY Jeong EM Bae JY Kang JJ Yang SJ Kim SW Noh DY Genomic copy number alterations as predictive markers of systemic recurrence in breast cancer Int J Cancer 2008 15;123 8 1807 15 10.1002/ijc.23672 18649361

[B18] Park JJ Kang JK Hong S Ryu ER Kim JI Lee JH Seo JS Genome-wide combination profiling of copy number and methylation offers an approach for deciphering misregulation and development in cancer cells Gene 2008 407 139 147 10.1016/j.gene.2007.10.011 17997235

[B19] Choi YW Choi JS Zheng LT Lim YJ Yoon HK Kim YH Wang YP Lim Y Comparative genomic hybridization array analysis and real time PCR reveals genomic alterations in squamous cell carcinomas of the lung Lung Cancer 2007 55 43 51 10.1016/j.lungcan.2006.09.018 17109992

[B20] Choi Y-W Bae SM Kim Y-W Lee HN Kim YW Park TC Ro DY Shin JC Shin SJ Seo JS Ahn W-S Gene expression profiles in squamous cell cervical carcinoma using array-based comparative genomic hybridization analysis Int J Gynecol Cancer 2007 17 687 96 10.1111/j.1525-1438.2007.00834.x 17504382

[B21] Pinkel D Straume T Gray JW Cytogenetic analysis using quantitative, high-sensitivity, fluorescence hybridization Proc Natl Acad Sci 1986 83 2934 38 3458254 10.1073/pnas.83.9.2934 PMC323421

[B22] Willenbrock H Fridlyand J A comparison study: applying segmentation to array CGH data for downstream analyses Bioinformatics 2005 21 4084 91 10.1093/bioinformatics/bti677 16159913

[B23] Lockwood WW Coe BP Williams AC MacAulay C Lam WL Whole genome tiling path array CGH analysis of segmental copy number alterations in cervical cancer cell lines Int J Cancer 2007 15;120 2 436 43 10.1002/ijc.22335 17096350

[B24] Lundgren K Holm K Nordenskjöld B Borg A Landberg G Gene products of chromosome 11q and their association with CCND1 gene amplification and tamoxifen resistance in premenopausal breast cancer Breast Cancer Res 2008 10 5 R81 18823530 10.1186/bcr2150 PMC2614516

[B25] Choi YW Choi JS Zheng LT Lim YJ Yoon HK Kim YH Wang YP Lim Y Comparative genomic hybridization array analysis and real time PCR reveals genomic alterations in squamous cell carcinomas of the lung Lung Cancer 2007 55 1 43 51 10.1016/j.lungcan.2006.09.018 17109992

[B26] Sy SM Wong N Lee TW Tse G Mok TS Fan B Pang E Johnson PJ Yim A Distinct patterns of genetic alterations in adenocarcinoma and squamous cell carcinoma of the lung Eur J Cancer 2004 40 1082 94 10.1016/j.ejca.2004.01.012 15093586

[B27] Höglund M Gisselsson D Hansen GB Mitelman F Statistical dissection of cytogenetic patterns in lung cancer reveals multiple modes of karyotypic evolution independent of histological classification Cancer Genet Cytogenet 2004 15 154 99 109 10.1016/j.cancergencyto.2004.01.030 15474144

[B28] Pardo J Torres W Martinez-Peñuela A Panizo A de Alava E García JL Pseudomesotheliomatous carcinoma of the lung with a distinct morphology, immunohistochemistry, and comparative genomic hybridization profile Ann Diagn Pathol 2007 11 241 51 10.1016/j.anndiagpath.2006.08.009 17630107

[B29] Yan WS Song LY Wei WD Li A Liang QW Liu JH Fang Y Chromosomal imbalance in primary lung squamous cell carcinoma and their relationship with smoking Ai Zheng 2005 24 47 52 15642199

[B30] Tong J Taylor P Jovceva E St-Germain JR Jin LL Nikolic A Gu X Li ZH Trudel S Moran MF Tandem Immunoprecipitation of Phosphotyrosine-Mass Spectrometry (TIPY-MS) Indicates C19ORF19 Becomes Tyrosine-Phosphorylated and Associated with Activated Epidermal Growth Factor Receptor J Proteome Res 2008 7 7 1067 77 10.1021/pr7006363 18271526

[B31] Hirsch FR Varella-Garcia M Bunn PA Jr Di Maria MV Veve R Bremmes RM Barón AE Zeng C Franklin WA Epidermal growth factor receptor in non-small-cell lung carcinomas: correlation between gene copy number and protein expression and impact on prognosis J Clin Oncol 2003 21 3798 807 10.1200/JCO.2003.11.069 12953099

[B32] Salomon DS Brandt R Ciardiello F Normanno N Epidermal growth factor-related peptides and their receptors in human malignancies Crit Rev Oncol Hematol 1995 19 182 232 10.1016/1040-8428(94)00144-I 7612182

[B33] Loukopoulos P Shibata T Katoh H Kokubu A Sakamoto M Yamazaki K Kosuge T Kanai Y Hosoda F Imoto I Ohki M Inazawa J Hirohashi S Genome-wide array-based comparative genomic hybridization analysis of pancreatic adenocarcinoma: identification of genetic indicators that predict patient outcome Cancer Sci 2007 98 392 400 10.1111/j.1349-7006.2007.00395.x 17233815 PMC11158398

[B34] Weiss MM Kuipers EJ Postma C Snijders AM Pinkel D Meuwissen SG Albertson D Meijer GA Genomic alterations in primary gastric adenocarcinomas correlate with clinicopathological characteristics and survival Cell Oncol 2004 26 307 17 15623941 10.1155/2004/454238 PMC4611111

[B35] Roque L Rodrigues R Pinto A Moura-Nunes V Soares J Chromosome imbalances in thyroid follicular neoplasms: a comparison between follicular adenomas and carcinomas Genes Chromosomes Cancer 2003 36 292 302 10.1002/gcc.10146 12557229

[B36] Okada S Tsuda H Takarabe T Yoshikawa H Taketani Y Hirohashi S Allelotype analysis of common epithelial ovarian cancers with special reference to comparison between clear cell adenocarcinoma with other histological types Jpn J Cancer Res 2002 93 798 806 12149146 10.1111/j.1349-7006.2002.tb01322.x PMC5927079

[B37] Heselmeyer K Macville M Schrock E Blegen H Hellstrom AC Shah K Auer G Ried T Advanced-stage cervical carcinomas are defined by a recurrent pattern of chromosomal aberrations revealing high genetic instability and a consistent gain of chromosome arm 3q Genes Chromosomes Cancer 1997 19 233 40 10.1002/(SICI)1098-2264(199708)19:4<233::AID-GCC5>3.0.CO;2-Y 9258658

[B38] Heselmeyer K Schrock E du Manoir S Blegen H Shah K Steinbeck R Auer G Ried T Gain of chromosome 3q defines the transition from severe dysplasia to invasive carcinoma of the uterine cervix Proc Natl Acad Sci USA 1996 93 479 84 8552665 10.1073/pnas.93.1.479 PMC40262

[B39] Yen CC Chen YJ Pan CC Lu KH Chen PC Hsia JY Chen JT Wu YC Hsu WH Wang LS Huang MH Huang BS Hu CP Chen PM Lin CH Copy number changes of target genes in chromosome 3q25.3-qter of esophageal squamous cell carcinoma: TP63 is amplified in early carcinogenesis but down-regulated as disease progressed World J Gastroentero 2005 7 11 1267 72 10.3748/wjg.v11.i9.1267 PMC4250671 15761962

[B40] Nanjundan M Nakayama Y Cheng KW Lahad J Liu J Lu K Kuo WL Smith-McCune K Fishman D Gray JW Mills GB Amplification of MDS1/EVI1 and EVI1, located in the 3q26.2 amplicon, is associated with favorable patient prognosis in ovarian cancer Cancer Res 2007 1 67 3074 84 10.1158/0008-5472.CAN-06-2366 17409414

[B41] Angulo B Suarez-Gauthier A Lopez-Rios F Medina PP Conde E Tang M Soler G Lopez-Encuentra A Cigudosa JC Sanchez-Cespedes M Expression signatures in lung cancer reveal a profile for EGFR-mutant tumours and identify selective PIK3CA overexpression by gene amplification J Pathol 2008 214 347 56 10.1002/path.2267 17992665

[B42] Tai AL Yan WS Fang Y Xie D Sham JS Guan XY Recurrent chromosomal imbalances in nonsmall cell lung carcinoma: the association between 1q amplification and tumor recurrence Cancer 2004 1;100 9 1918 27 10.1002/cncr.20190 15112273

[B43] Lukashova-v Zangen I Kneitz S Monoranu CM Rutkowski S Hinkes B Vince GH Huang B Roggendorf W Ependymoma gene expression profiles associated with histological subtype, proliferation, and patient survival Acta Neuropathol 2007 113 3 325 37 10.1007/s00401-006-0190-5 17265049

[B44] Liu W Ahmad SA Jung YD Reinmuth N Fan F Bucana CD Ellis LM Coexpression of ephrin-Bs and their receptors in colon carcinoma Cancer 2002 15;94 4 934 9 10.1002/cncr.10122 11920461

[B45] Kuraya M Matsushita M Endo Y Thiel S Fujita T Expression of H-ficolin/Hakata antigen, mannose-binding lectin-associated serine protease (MASP)-1 and MASP-3 by human glioma cell line T98G Int Immunol 2003 15 1 109 17 10.1093/intimm/dxg008 12502731

